# Supplementary material for: Identification of photoperiod-induced specific miRNAs in the adrenal glands of Sunite sheep (Ovis aries)
Source: Front Vet Sci. 2022 Jul 22;9:888207. doi: 10.3389/fvets.2022.888207 (PMC9354845; doi:10.3389/fvets.2022.888207)
Supplement: Supplementary file 1 [file Data_Sheet_1.ZIP › Supplementary file/miRDeep2 file.zip!/miRDeep2 file/Know_Structure/LP42A3/oar-mir-3959.pdf]

miRBase precursor : oar-mir-3959
Total read count : 30122
oar-miR-3959-5p read count : 27145
oar-miR-3959-3p read count : 2976
remaining reads : 1

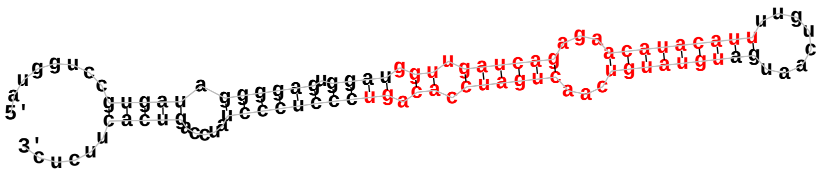

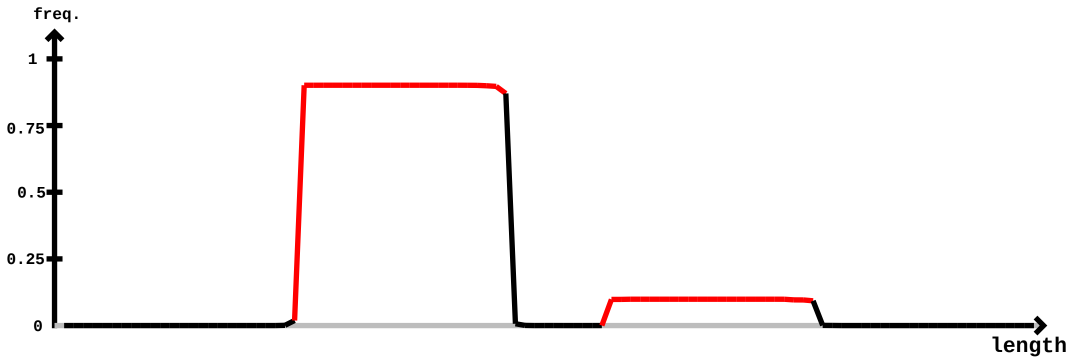

oar-miR-3959-5p

oar-miR-3959-3p

```
5'- augguccgugauaggggaguggauggguugaucagagaacauacauuuugucaaugauguaugucaacugauccacagucccucccuauccagucacuucuc   -3'   exp
    .......(((((.((((((.((((.((.((((((...(((((((((........)))))))))...)))))).)).))))))))))).....))))).....   reads   mm   sample
    ......................augguugaucagagaacau............................................................   1       0    seq
    ......................augguugaucagagaacaua...........................................................   4       0    seq
    ......................augguugaucagagaacauac..........................................................   9       0    seq
    ......................augguugaucagagaacauaca.........................................................   13      0    seq
    .......................ugguugaucagagaacaua...........................................................   1       0    seq
    .......................ugguugaucagagaacauac..........................................................   1       0    seq
    .......................ugguugaucagagaacauaca.........................................................   8       0    seq
    .......................ugguugaucagagaacauacau........................................................   24      0    seq
    .......................ugguugaucagagaacauacauu.......................................................   508     0    seq
    .......................ugguugaucagagaacauacauuu......................................................   4       0    seq
    ........................gguugaucagagaacau............................................................   4       0    seq
    ........................gguugaucagagaacaua...........................................................   4       0    seq
    ........................gguugaucagagaacauac..........................................................   35      0    seq
    ........................gguugaucagagaacauaca.........................................................   52      0    seq
    ........................gguugaucagagaacauacau........................................................   773     0    seq
    ........................gguugaucagagaacauacauu.......................................................   25500   0    seq
    ........................gguugaucagagaacauacauuu......................................................   183     0    seq
    ........................gguugaucagagaacauacauuuu.....................................................   17      0    seq
    ........................gguugaucagagaacauacauuuuguc..................................................   1       0    seq
    .........................guugaucagagaacauacauu.......................................................   2       0    seq
    ..........................uugaucagagaacauacauuuu.....................................................   1       0    seq
    .................................................ucaaugauguaugucaacugauc.............................   1       0    seq
    .......................................................auguaugucaacugauccacagu.......................   1       0    seq
    ........................................................uguaugucaacugaucc............................   1       0    seq
    ........................................................uguaugucaacugaucca...........................   2       0    seq
    ........................................................uguaugucaacugauccac..........................   72      0    seq
    ........................................................uguaugucaacugauccaca.........................   9       0    seq
    ........................................................uguaugucaacugauccacag........................   98      0    seq
    ........................................................uguaugucaacugauccacagu.......................   2764    0    seq
    ........................................................uguaugucaacugauccacaguc......................   5       0    seq
    .........................................................guaugucaacugauccacagu.......................   3       0    seq
    ..........................................................uaugucaacugauccacagu.......................   4       0    seq
    ..........................................................uaugucaacugauccacaguc......................   5       0    seq
    ..........................................................uaugucaacugauccacagucc.....................   12      0    seq
```

oar-miR-3959-5p

oar-miR-3959-3p

augguccgugauaggggaguggauggguugaucagagaacauacauuuugucaaugauguaugucaacugauccacagucccucccuauccagucacuucuc
